# Supplementary material for: Virtual birefringence imaging and histological staining of amyloid deposits in label-free tissue using autofluorescence microscopy and deep learning
Source: Nat Commun. 2024 Sep 12;15:7978. doi: 10.1038/s41467-024-52263-z (PMC11393327; doi:10.1038/s41467-024-52263-z)
Supplement: Supplementary file 1 — Supplementary Information [file 41467_2024_52263_MOESM1_ESM.pdf]

Supplementary Information for:

Virtual birefringence imaging and histological staining of amyloid deposits in label-free tissue using autofluorescence microscopy and deep learning

Authors

Xilin Yang[1,2,3], Bijie Bai[1,2,3], Yijie Zhang[1,2,3], Musa Aydin[1,4], Yuzhu Li[1,2,3], Sahan Yoruc Selcuk[1,2,3], Paloma Casteleiro Costa[1,2,3] Zhen Guo[1], Gregory A. Fishbein[5], Karine Atlan[6], William Dean Wallace[7], Nir Pillar[1,2,3*], Aydogan Ozcan[1,2,3,8*]

Affiliations

[1]Electrical and Computer Engineering Department, University of California, Los Angeles, CA, 90095, USA.

[2]Bioengineering Department, University of California, Los Angeles, CA, 90095, USA.

[3]California NanoSystems Institute (CNSI), University of California, Los Angeles, CA, 90095, USA.

[4]Department of Computer Engineering, Fatih Sultan Mehmet Vakif University, Istanbul, 34038, Turkiye

[5]Department of Pathology and Laboratory Medicine, David Geffen School of Medicine at the University of California, Los Angeles, CA, 90095, USA.

[6]Department of Pathology, Hadassah Hebrew University Medical Center, Jerusalem, 91120, Israel

[7]Department of Pathology, Keck School of Medicine, University of Southern California, Los Angeles, CA, 90033, USA.

[8]Department of Surgery, University of California, Los Angeles, CA, 90095, USA.

[*]Correspondence to: Aydogan Ozcan, ozcan@ucla.edu; Nir Pillar, npillar@g.ucla.edu

Contents

Supplementary Figure 1. Congo Red Virtual Staining: Training and Testing.

Supplementary Figure 2. Data preparation and the workflow of pathologists' evaluations.

Supplementary Figure 3. Examples of image patches and pathologist scores for bright-field Congo red stain image quality.

Supplementary Figure 4. Paired comparison of brightfield image quality.

Supplementary Figure 5. Example fields-of-view with scores for M5-M7. All images are selected from the training dataset.

Supplementary Figure 6. Confusion matrices for the birefringence image quality scores of pathologists (P1, P2 and P3) blindly comparing virtually stained and histochemically stained images.

Supplementary Figure 7. Examples of larger image patches and pathologist scores for birefringence image quality.

Supplementary Figure 8. Additional examples of larger image patches and pathologist scores for birefringence image quality.

Supplementary Figure 9. Workflow for calculating quantitative metrics used for the comparison of histochemically and virtually stained Congo-red images.

Supplementary Figure 10. Color histograms in YCbCr color space for the entire test dataset.

Supplementary Figure 11. Quantitative evaluation results for comparing histochemically and virtually stained brightfield Congo-red images.

Supplementary Figure 12. Transfer learning for a sensor with a higher noise level.

Supplementary Figure 13. Ablation study with different numbers of input channels.

Supplementary Table 1. Average values and standard deviations of the pathologists' evaluation scores.

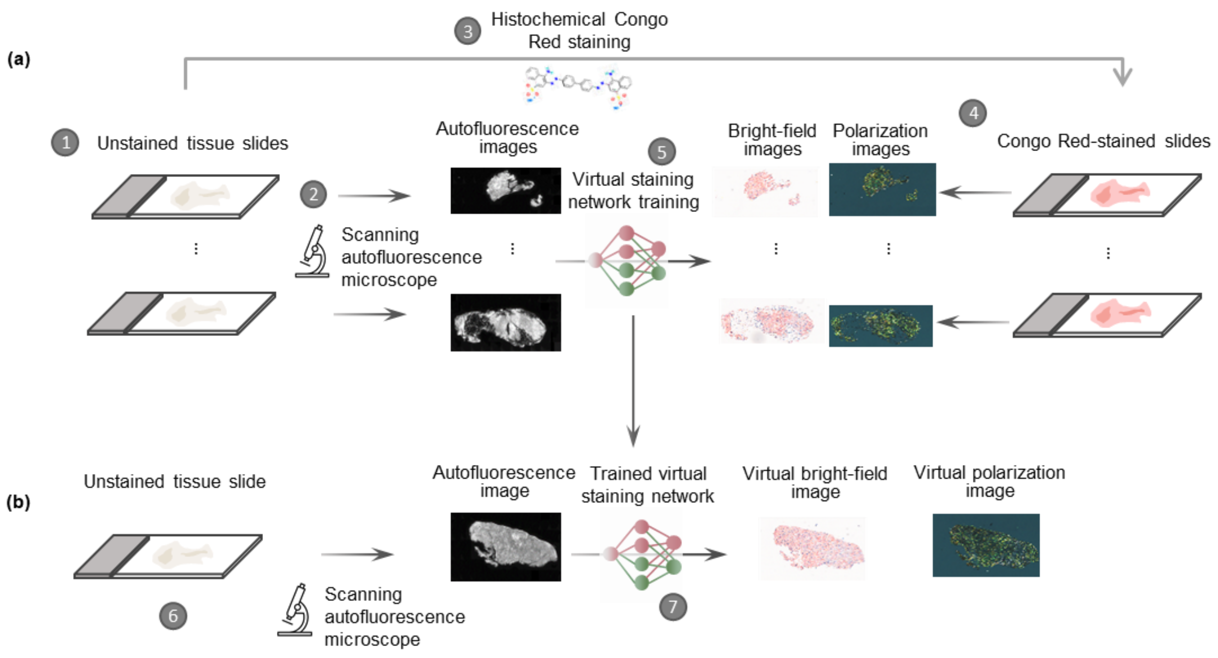

Supplementary Figure 1. Congo Red Virtual Staining: Training and Testing. (a) In the traditional pathology workflow, unstained slides suspected of having amyloid deposits undergo Congo red staining. A pathologist examines the Congo red-stained slides, and if pink-salmon areas are identified, these areas are visualized under polarized light microscopy to provide a definitive diagnosis (1→3→4). For the virtual Congo red training step, unstained slides undergo autofluorescence scanning followed by Congo red histochemical staining (the latter used as ground truth). The Congo red-stained slides are then scanned using brightfield and polarized microscopy. By training a deep neural network-based algorithm, our method learns to transform the unstained/label-free slide into a virtual Congo red stain, in both its brightfield and polarization image channels (1→2→3→4→5). (b) During the model evaluation phase, unstained slides are converted into virtual Congo red-stained slides and sent for pathologist assessment. Chemical structure of Congo red obtained from https://pubchem.ncbi.nlm.nih.gov/compound/11313 #section=2D-Structure.

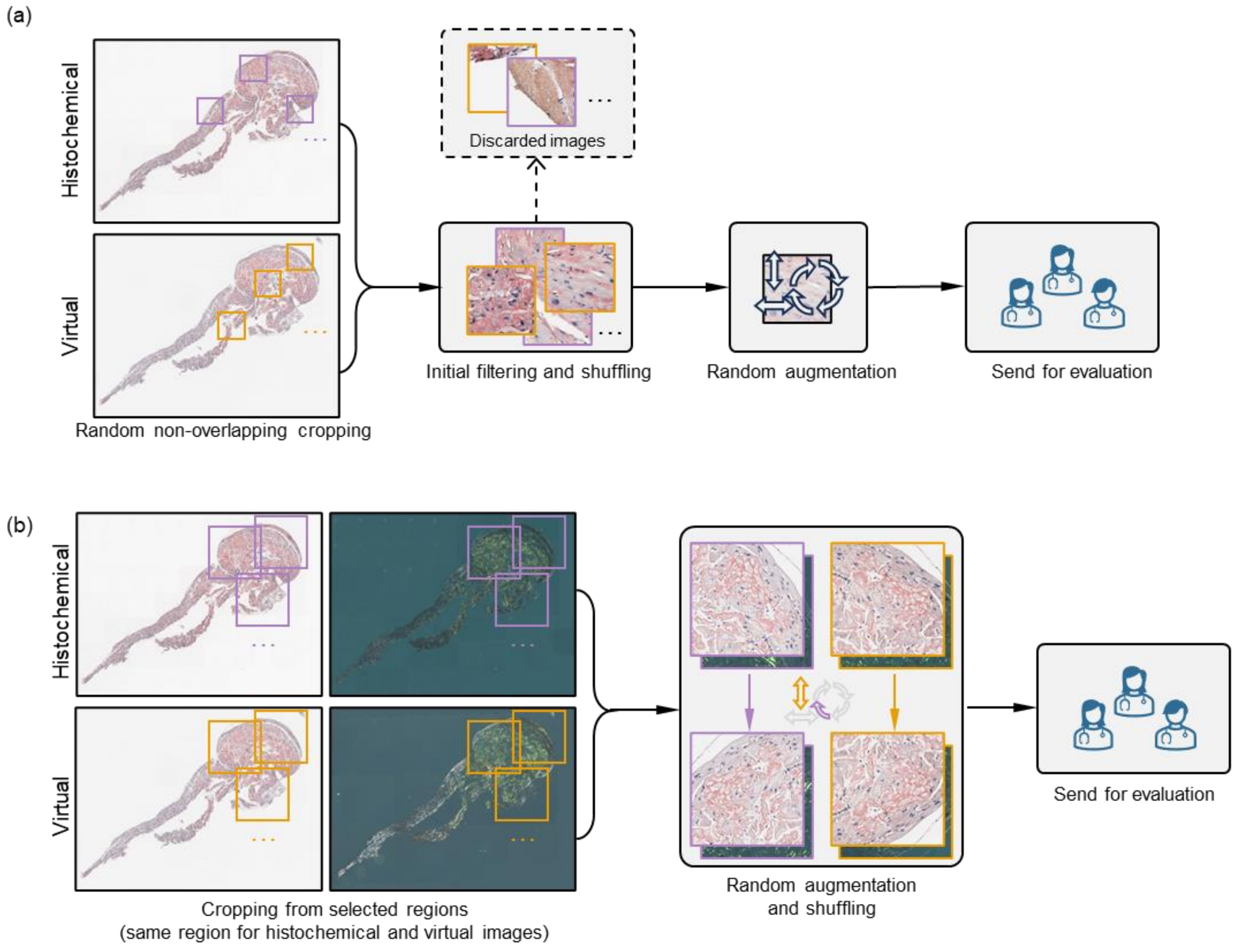

Supplementary Figure 2. **Data preparation and the workflow of pathologists' evaluations.** (a) Workflow for part 1 evaluation. (b) Workflow for part 2 evaluation.

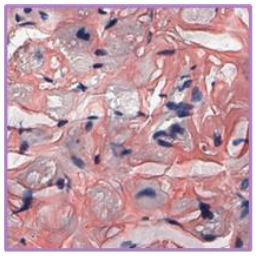

Histochemical FOV 1 score table

	M1	M2	M3	M4
P1	3	3	3	3
P2	4	4	4	4
P3	4	4	4	4

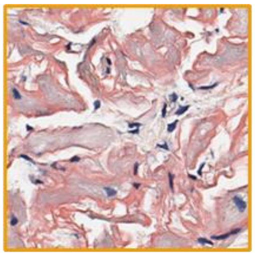

Virtually stained FOV 1 score table

	M1	M2	M3	M4
P1	3	2	3	3
P2	3	2	2	4
P3	3	2	3	3

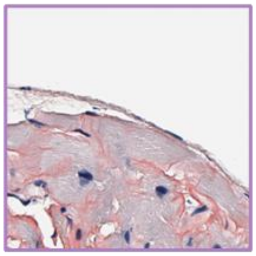

Histochemical FOV 2 score table

	M1	M2	M3	M4
P1	3	3	3	3
P2	4	4	4	4
P3	3	2	1	3

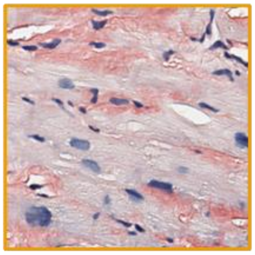

Virtually stained FOV 2 score table

	M1	M2	M3	M4
P1	3	3	3	3
P2	4	4	4	4
P3	3	3	3	3

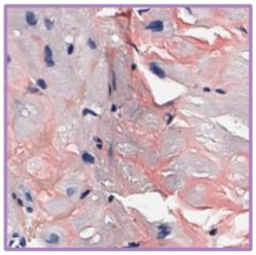

Histochemical FOV 3 score table

	M1	M2	M3	M4
P1	2	1	1	2
P2	4	/	4	3
P3	3	3	3	4

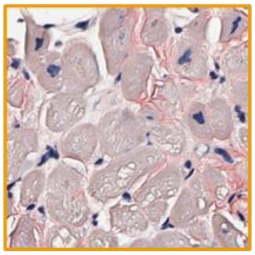

Virtually stained FOV 3 score table

	M1	M2	M3	M4
P1	3	3	3	3
P2	4	4	4	4
P3	3	3	2	2

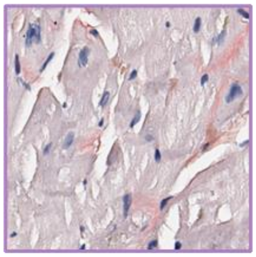

Histochemical FOV 4 score table

	M1	M2	M3	M4
P1	3	3	3	2
P2	3	3	3	3
P3	3	3	2	1

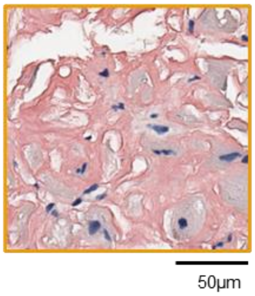

Virtually stained FOV 4 score table

	M1	M2	M3	M4
P1	2	3	3	3
P2	3	3	3	3
P3	3	3	2	3

Supplementary Figure 3. Examples of image patches and pathologist scores for bright-field Congo red stain image quality.

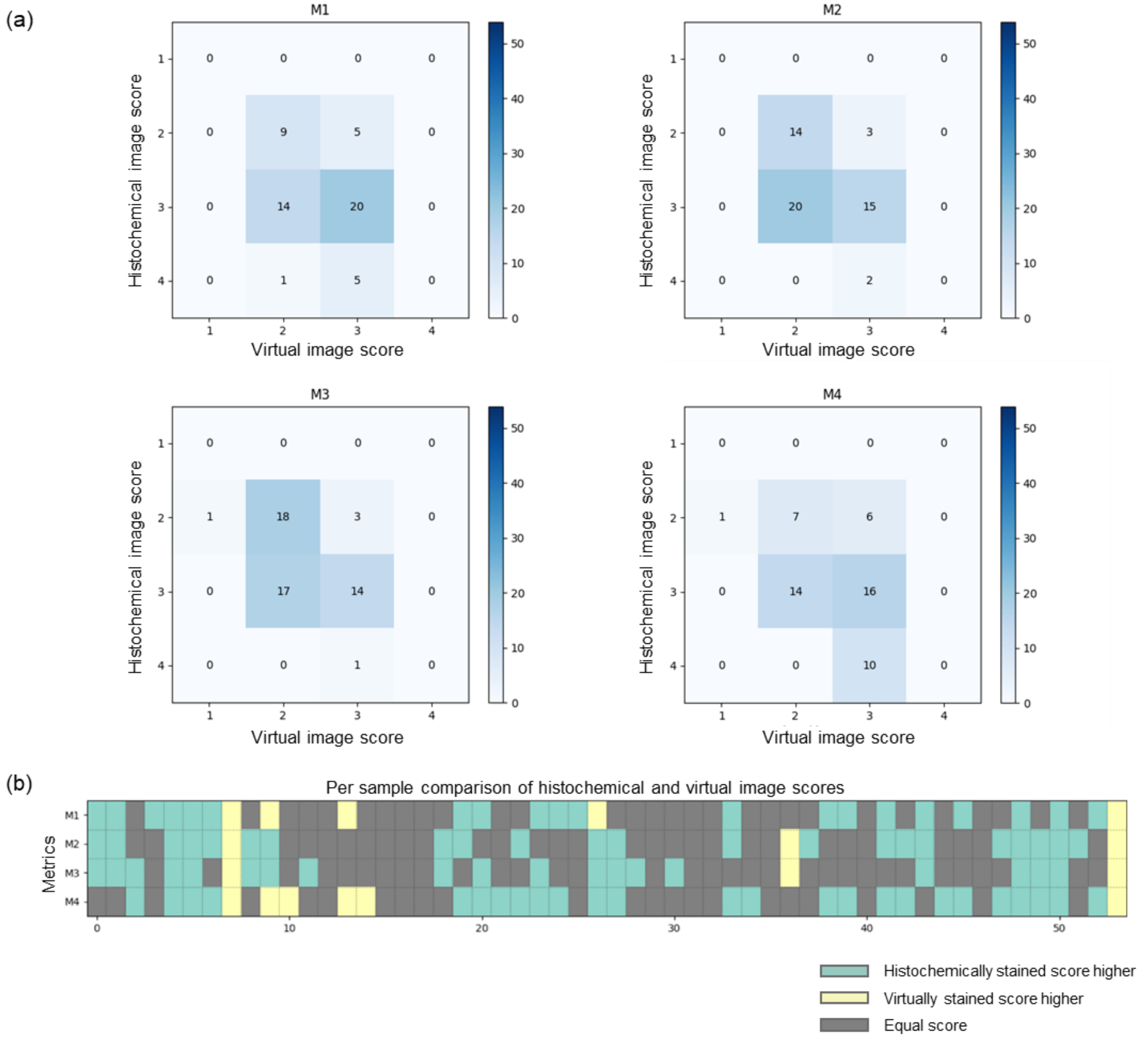

Supplementary Figure 4. **Paired comparison of brightfield image quality.** (a) Confusion matrices for the bright-field image quality scores of pathologists (P1, P2 and P3) blindly comparing virtually stained and histochemically stained images. (b) Per sample comparison of histochemical and virtually stained image scores.

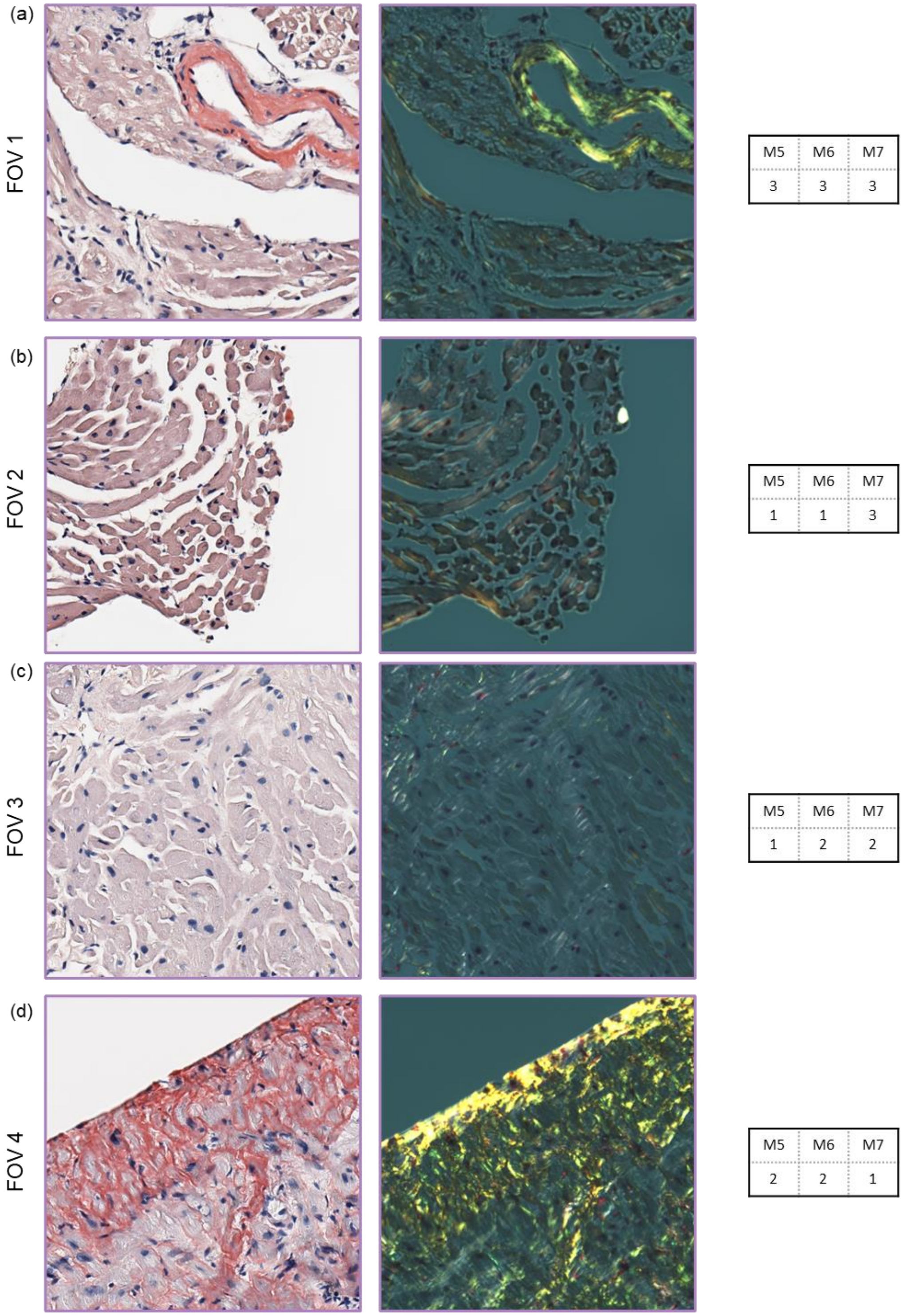

Supplementary Figure 5. Four example fields-of-views (a-d) with scores for M5-M7. All images are selected from the training dataset.

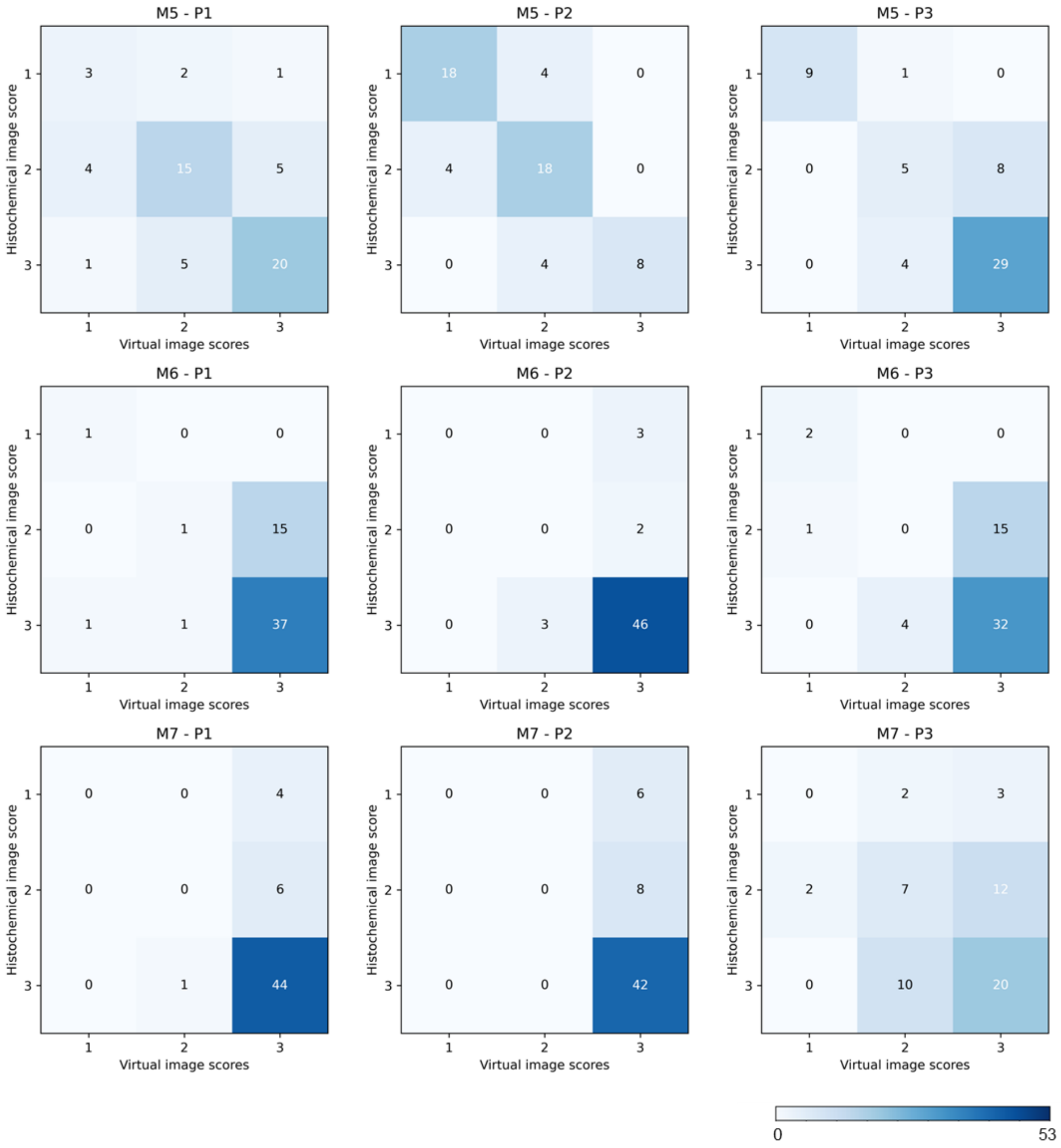

Supplementary Figure 6. **Confusion matrices for the birefringence image quality scores of pathologists (P1, P2 and P3) blindly comparing virtually stained and histochemically stained images.**

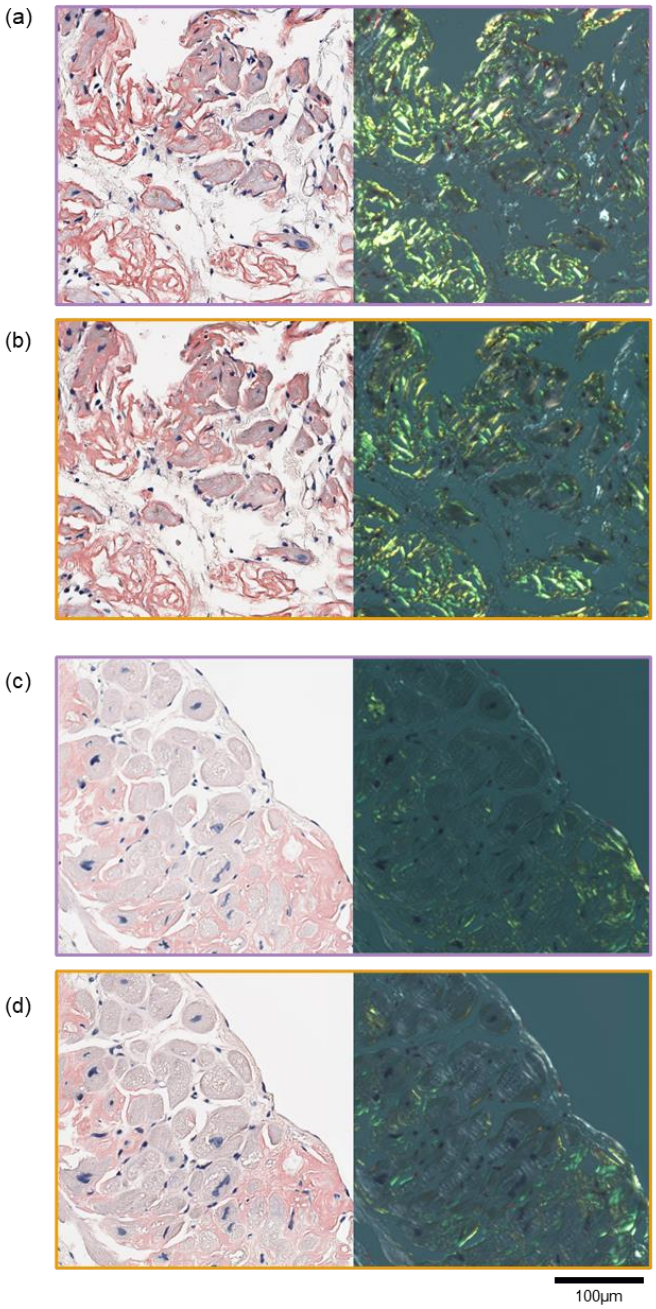

Histochemically stained
FOV 1 score table

	M5	M6	M7
P1	2	3	3
P2	2	3	3
P3	3	3	3

Virtually stained
FOV 1 score table

	M5	M6	M7
P1	3	2	3
P2	2	3	3
P3	3	3	2

Histochemically stained
FOV 2 score table

	M5	M6	M7
P1	2	3	3
P2	2	3	3
P3	2	2	3

Virtually stained
FOV 2 score table

	M5	M6	M7
P1	1	3	3
P2	1	3	3
P3	3	3	2

Supplementary Figure 7. **Representative larger image patches and pathologist scores for brightfield birefringence image quality.** Panels (a) and (b) display histochemical and virtually stained images, respectively, for the first field-of-view (FOV). Panels (c) and (d) present the corresponding images for the second FOV.

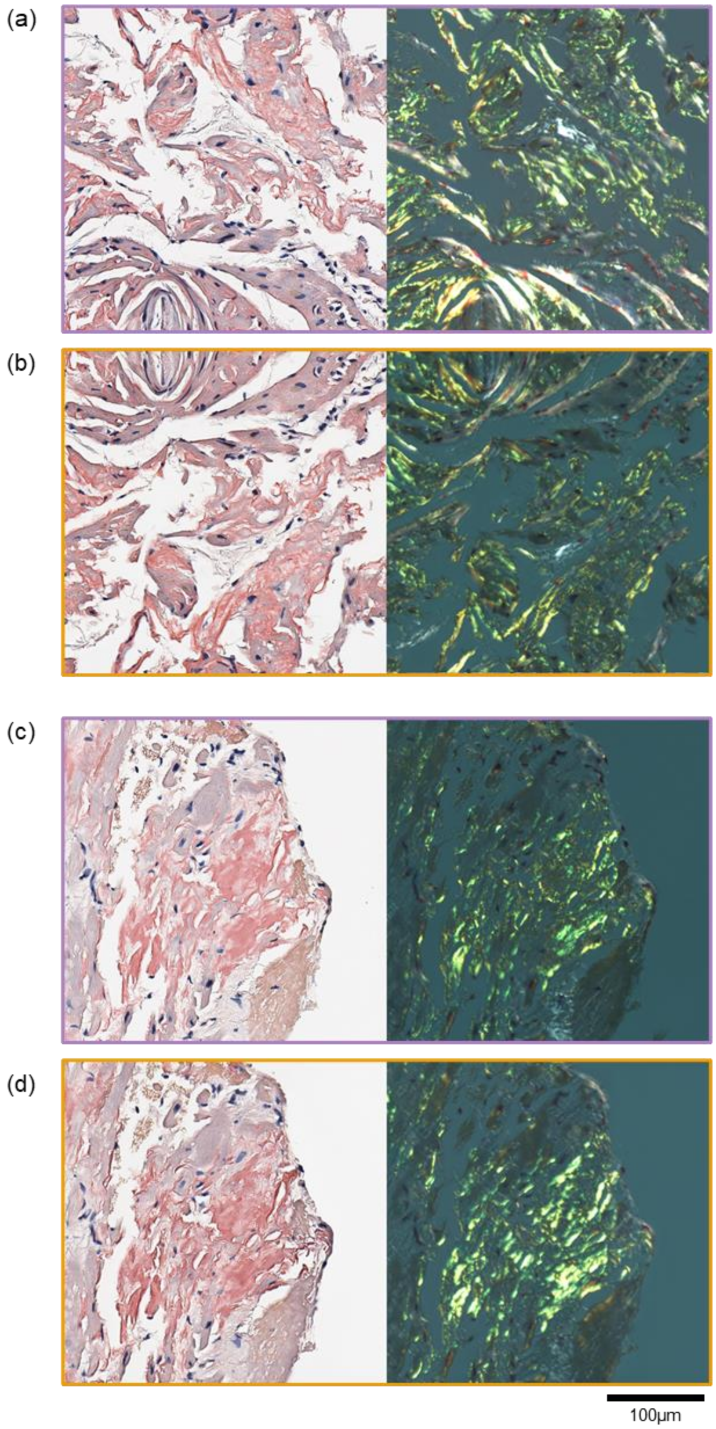

Histochemically stained
FOV 1 score table

	M5	M6	M7
P1	3	2	3
P2	1	3	3
P3	2	3	3

Virtually stained
FOV 1 score table

	M5	M6	M7
P1	1	3	3
P2	1	2	3
P3	3	3	2

Histochemically stained
FOV 2 score table

	M5	M6	M7
P1	2	3	3
P2	3	3	3
P3	3	3	3

Virtually stained
FOV 2 score table

	M5	M6	M7
P1	2	3	3
P2	3	3	3
P3	3	3	3

Supplementary Figure 8. Additional examples of larger image patches and corresponding pathologist scores for brightfield and birefringence image quality. Panels (a) and (b) showcase virtually stained images with inferior quality scores. Panels (c) and (d) present virtually stained images with equivalent quality scores.

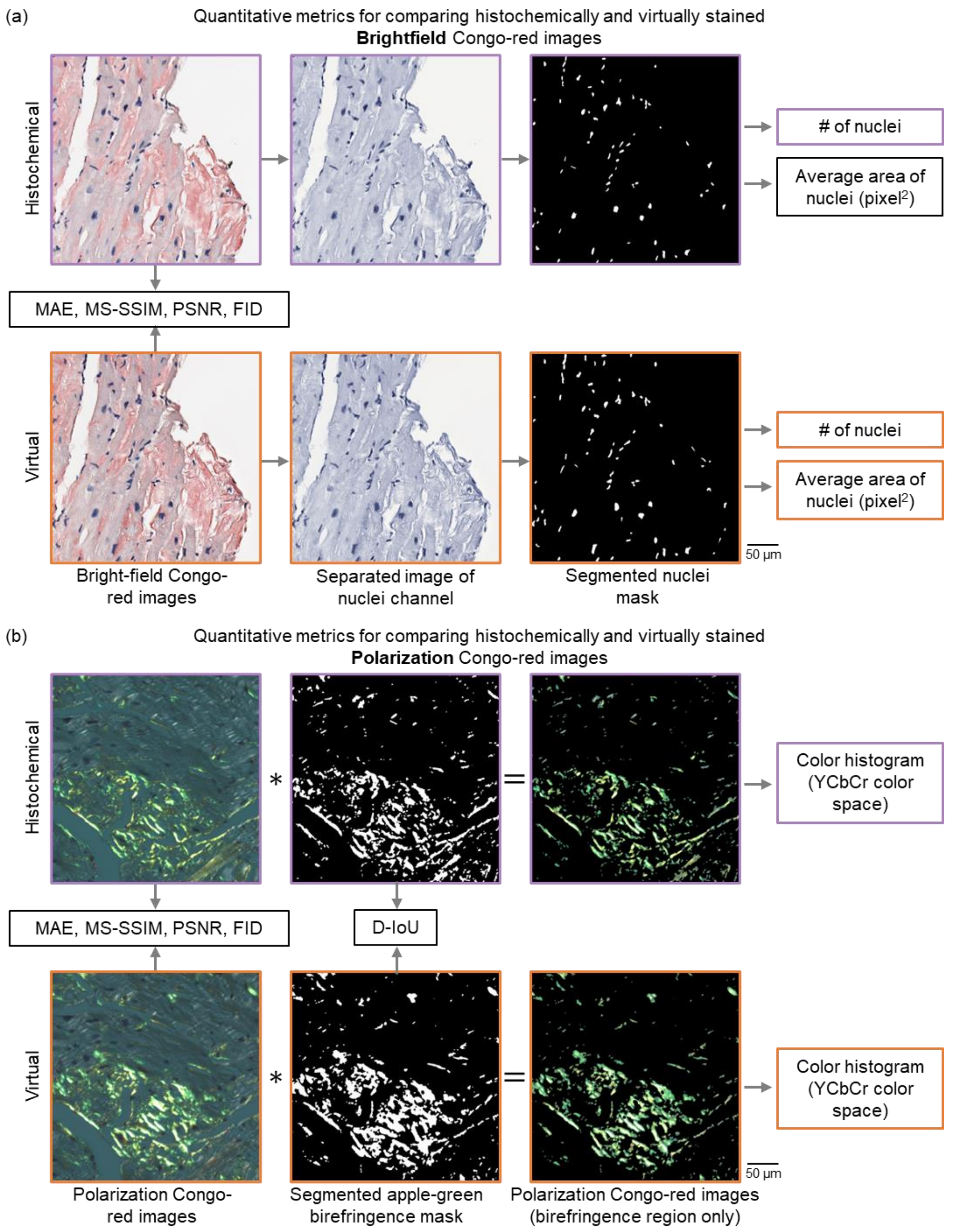

Supplementary Figure 9. **Workflow for calculating quantitative metrics used for the comparison of histochemically and virtually stained Congo-red images.** (a) The workflow of quantifying various metrics for histochemical and virtual brightfield Congo-red images, including mean absolute error (MAE), multiscale structural similarity index metric (MS-SSIM), peak signal-to-noise ratio (PSNR), Fréchet inception distance (FID), the number of nuclei per field-of-view (FOV) and the average area of nuclei. (b) The workflow of quantifying various metrics for histochemical and virtual polarization Congo-red images, including MAE, MS-SSIM, PSNR, FID, down-sampled intersection-over-union (D-IoU) between segmented apple-green birefringence masks (histochemical and virtual), color histograms in YCbCr color space for the whole FOV and the birefringence regions only.

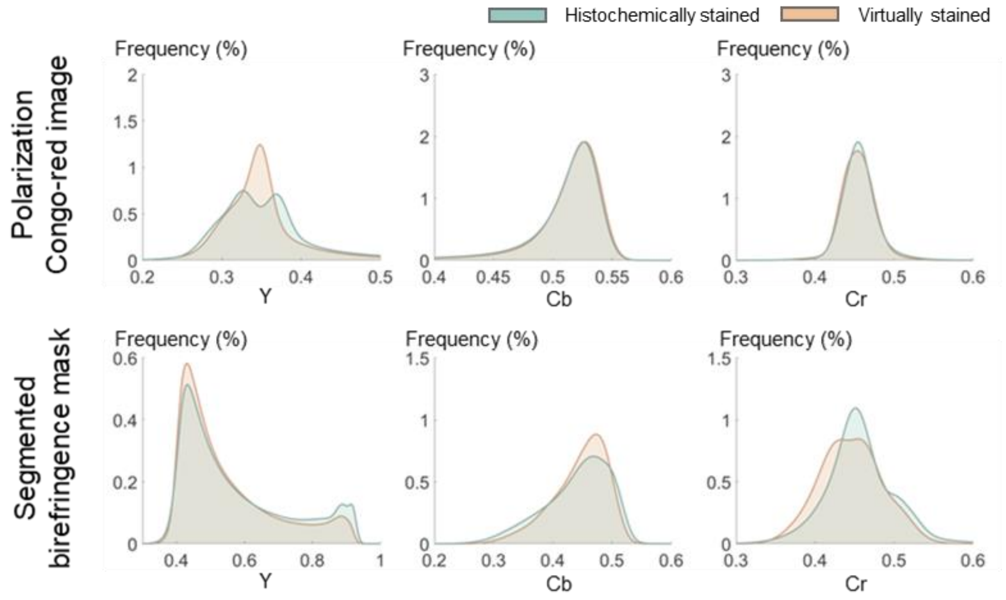

Supplementary Figure 10. Color histograms in YCbCr color space for the entire test dataset. The YCbCr color histograms were obtained from all the test image fields-of-view (FOVs). The blue curves represent the distributions of the histochemically stained images, while the red ones present the virtually stained images.

(a)

MAE	0.0741 ± 0.0154	MS-SSIM	0.7096 ± 0.1050
PSNR	18.7105 ± 1.4135	FID	80.5328

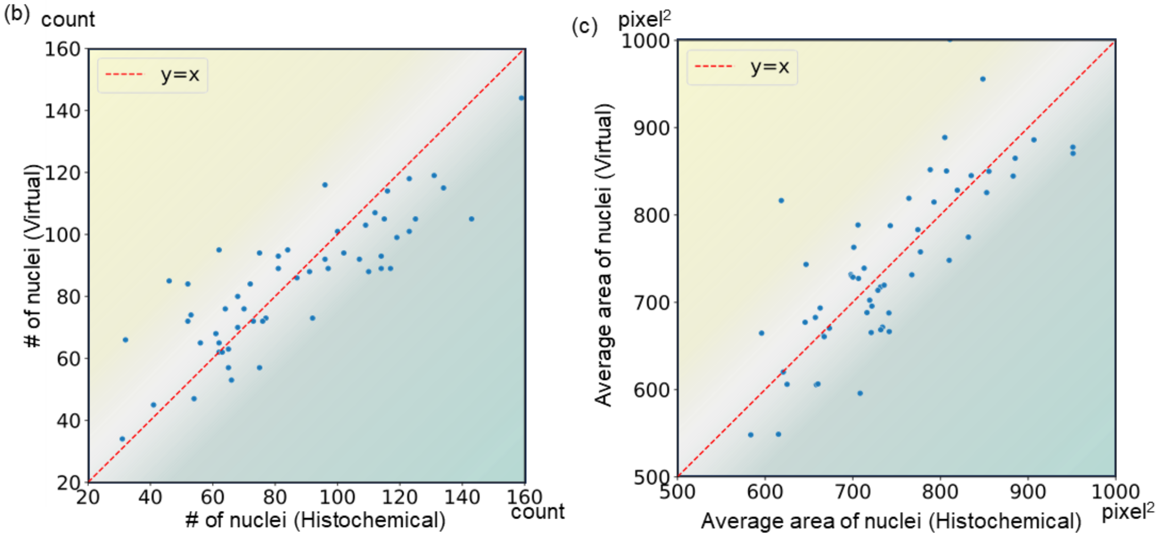

Supplementary Figure 11. **Quantitative evaluation results for comparing histochemically and virtually stained brightfield Congo-red images.** (a) The table listing mean absolute error (MAE), multiscale structural similarity index metric (MS-SSIM), peak signal-to-noise ratio (PSNR), and Fréchet inception distance (FID) between histochemically and virtually stained brightfield Congo-red images. (b) The number of nuclei per field-of-view (FOV) within histochemically stained brightfield Congo-red images vs. the number of nuclei per FOV within virtually stained brightfield Congo-red images. (c) The average area of nuclei within histochemically stained brightfield Congo-red images vs. the average area of nuclei within virtually stained brightfield Congo-red images.

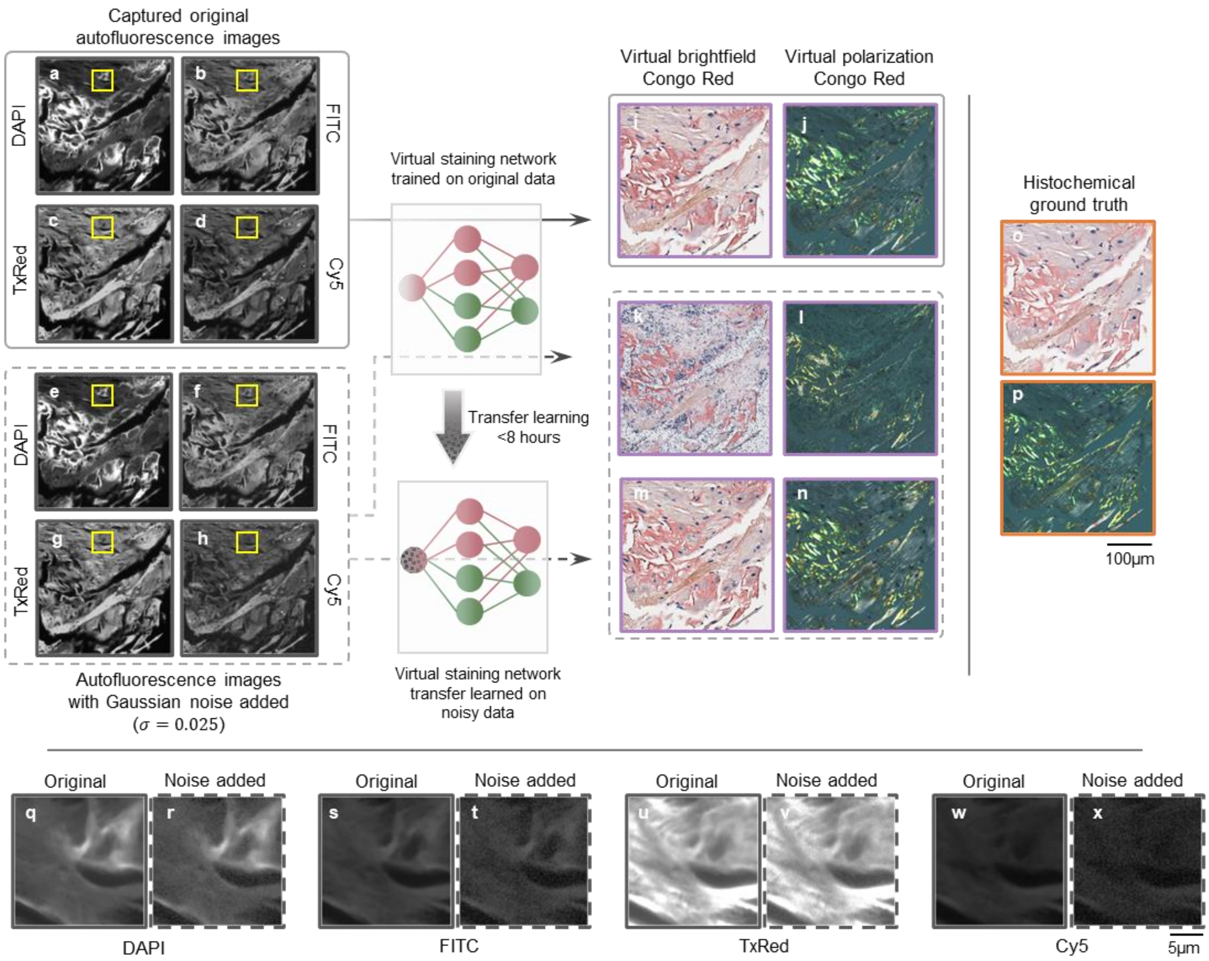

Supplementary Figure 12. **Transfer learning for a sensor with a higher noise level.** (a-d) Autofluorescence images as captured using different fluorescence channels. (e-h) Autofluorescence images with added Gaussian noise (σ=0.025). (i-j) Virtual Congo red images from the model trained and tested with the original autofluorescence images. (k-l) Virtual Congo red images from the model trained with original autofluorescence images and tested with autofluorescence images that include additional Gaussian noise. (m-n) Virtual Congo red images from the model trained and tested with autofluorescence images containing added Gaussian noise. (o-p) The corresponding histochemical brightfield and polarization microscopy ground truths. (q-x) Zoomed-in regions highlighted in (a-h), showing the differences introduced by the added noise.

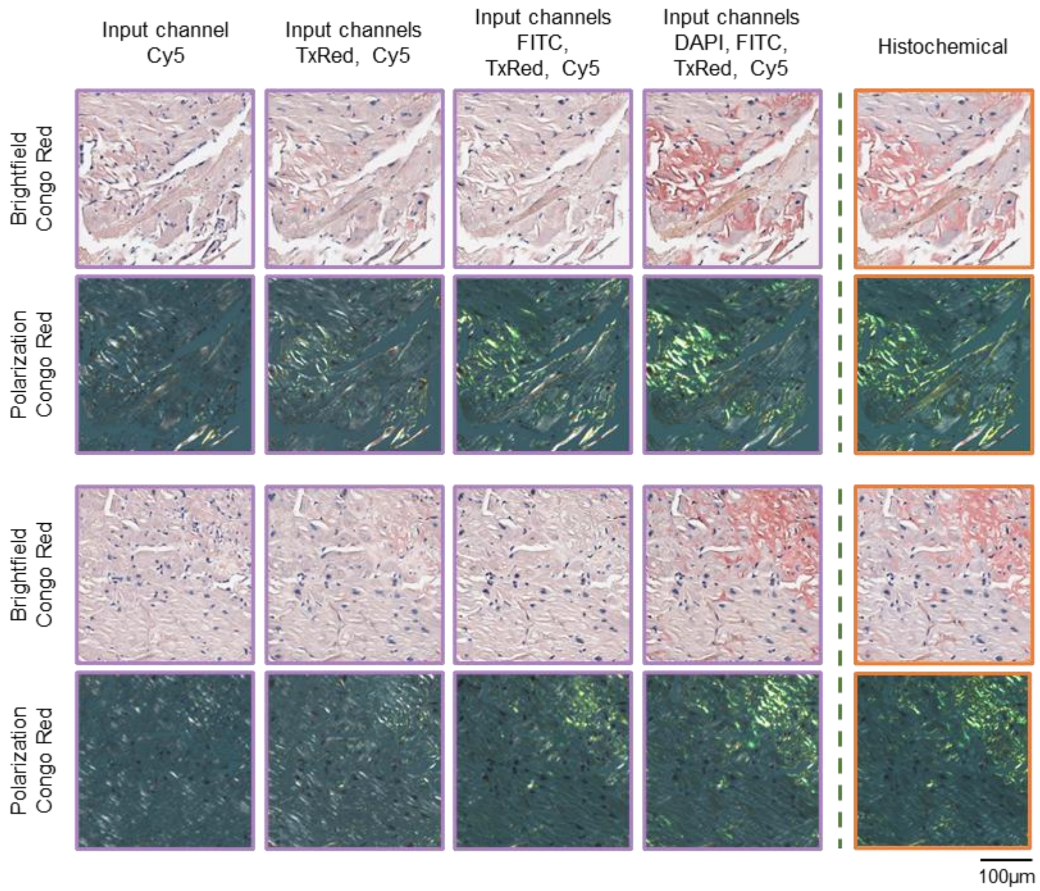

Supplementary Figure 13. **Ablation study with different numbers of input channels.** Virtual brightfield and polarization Congo red images inferred by the network that was trained and tested with 1 channel (Cy5), 2 channels (TxRed and Cy5), 3 channels (FITC, TxRed and Cy5), and 4 channels (DAPI, FITC, TxRed, and Cy5) of autofluorescence images. The histochemical staining is shown in the last column for comparison.

Histochemically stained | Virtually stained

	M1		M2		M3		M4		M5		M6		M7	
**P1**	2.67 (0.51)	2.58 (0.55)	2.64 (0.56)	2.48 (0.55)	2.45 (0.65)	2.30 (0.59)	2.41 (0.78)	2.44 (0.73)	2.36 (0.67)	2.32 (0.71)	2.68 (0.5)	2.89 (0.41)	2.75 (0.58)	2.98 (0.13)
**P2**	3.28 (0.63)	3.03 (0.65)	3.09 (0.72)	2.75 (0.75)	3.01 (0.7)	2.83 (0.69)	3.21 (0.65)	3.17 (0.52)	1.82 (0.76)	1.75 (0.69)	2.82 (0.54)	2.94 (0.23)	2.64 (0.67)	3.00 (0.0)
**P3**	3.08 (0.71)	2.74 (0.72)	2.83 (0.72)	2.32 (0.75)	2.68 (0.71)	2.67 (0.63)	2.51 (0.91)	2.32 (0.8)	2.41 (0.77)	2.5 (0.76)	2.63 (0.55)	2.8 (0.51)	2.45 (0.65)	2.59 (0.56)

average
(standard deviation)

Supplementary Table 1. **Average values and standard deviations of the pathologists' evaluation scores.** M1-M7: metrics 1 to 7. P1-P3: pathologist 1 to 3.
