# Supplementary material for: Virtual birefringence imaging and histological staining of amyloid deposits in label-free tissue using autofluorescence microscopy and deep learning
Source: Nat Commun. 2024 Sep 12;15:7978. doi: 10.1038/s41467-024-52263-z (PMC11393327; doi:10.1038/s41467-024-52263-z)
Supplement: Supplementary file 2 — Reporting Summary [file 41467_2024_52263_MOESM2_ESM.pdf]

nature portfolio

Corresponding author(s): Aydogan Ozcan

Last updated by author(s): Aug 18, 2024

Reporting Summary

Nature Portfolio wishes to improve the reproducibility of the work that we publish. This form provides structure for consistency and transparency in reporting. For further information on Nature Portfolio policies, see our Editorial Policies and the Editorial Policy Checklist.

Statistics

For all statistical analyses, confirm that the following items are present in the figure legend, table legend, main text, or Methods section.

n/a	Confirmed	
☐	☒	The exact sample size (n) for each experimental group/condition, given as a discrete number and unit of measurement
☐	☒	A statement on whether measurements were taken from distinct samples or whether the same sample was measured repeatedly
☒	☐	The statistical test(s) used AND whether they are one- or two-sided<br>*Only common tests should be described solely by name; describe more complex techniques in the Methods section.*
☒	☐	A description of all covariates tested
☒	☐	A description of any assumptions or corrections, such as tests of normality and adjustment for multiple comparisons
☐	☒	A full description of the statistical parameters including central tendency (e.g. means) or other basic estimates (e.g. regression coefficient) AND variation (e.g. standard deviation) or associated estimates of uncertainty (e.g. confidence intervals)
☒	☐	For null hypothesis testing, the test statistic (e.g. F, t, r) with confidence intervals, effect sizes, degrees of freedom and P value noted<br>*Give P values as exact values whenever suitable.*
☒	☐	For Bayesian analysis, information on the choice of priors and Markov chain Monte Carlo settings
☒	☐	For hierarchical and complex designs, identification of the appropriate level for tests and full reporting of outcomes
☒	☐	Estimates of effect sizes (e.g. Cohen's d, Pearson's r), indicating how they were calculated

Our web collection on statistics for biologists contains articles on many of the points above.

Software and code

Policy information about availability of computer code

Data collection	Autofluorescence images of label-free tissue samples were taken using a conventional scanning fluorescence microscope (IX-83, Olympus) equipped with a ×40/0.95NA objective lens (UPLSAPO, Olympus) managed by µManager (version 1.4) software. Brightfield images were obtained using a scanning microscope (AxioScan Z1, Zeiss) with a ×20/0.8NA objective lens (Plan-Apo). Polarized images of Congo red-stained slides were captured using a modified conventional brightfield microscope (IX-83, Olympus) with a halogen lamp used as the illumination source without blue filters. The microscope was equipped with a linear polarizer (U-POT, Olympus) and an analyzer with an adjustable wave plate (U-GAN, Olympus).
Data analysis	All the image processing and dataset generation were performed using MATLAB version R2023a (Mathworks) and Fiji version 2.9.0 (https://imagej.net/software/fiji/). The neural networks were implemented and trained using Python version 3.12.0 and PyTorch version 1.9.0 with CUDA toolkit version 11.8. The codes for our virtual staining framework can be found at: https://github.com/Mikeeeeeey/AmylVirtualStain

For manuscripts utilizing custom algorithms or software that are central to the research but not yet described in published literature, software must be made available to editors and reviewers. We strongly encourage code deposition in a community repository (e.g. GitHub). See the Nature Portfolio guidelines for submitting code & software for further information.

Data

Policy information about availability of data

All manuscripts must include a data availability statement. This statement should provide the following information, where applicable:

- Accession codes, unique identifiers, or web links for publicly available datasets
- A description of any restrictions on data availability
- For clinical datasets or third party data, please ensure that the statement adheres to our policy

All data supporting the results of this study are available withn the main text and Supplementary Information.
Heart tissue sections were obtained from USC Keck School of Medicine under IRB #HS-20-00151. The codes for our virtual staining framework can be found at: https://github.com/Mikeeeeeey/AmylVirtualStain

Research involving human participants, their data, or biological material

Policy information about studies with human participants or human data. See also policy information about sex, gender (identity/presentation), and sexual orientation and race, ethnicity and racism.

Reporting on sex and gender	Since the tissue slides data that we used for this study were all de-identified and obtained from existing specimen under IRB approval (IRB #HS-20-00151), we are unable to provide the information such as sex, gender, race, ethnicity, and other aspects.
Reporting on race, ethnicity, or other socially relevant groupings	Since the tissue slides data that we used for this study were all de-identified and obtained from existing specimen under IRB approval (IRB #HS-20-00151), we are unable to provide the information such as sex, gender, race, ethnicity, and other aspects.
Population characteristics	N/A
Recruitment	N/A
Ethics oversight	The tissue slides data that we used for this study were all de-identified and obtained from existing specimen under IRB approval (IRB #HS-20-00151, USC Keck School of Medicine).

Note that full information on the approval of the study protocol must also be provided in the manuscript.

Field-specific reporting

Please select the one below that is the best fit for your research. If you are not sure, read the appropriate sections before making your selection.

☒ Life sciences ☐ Behavioural & social sciences ☐ Ecological, evolutionary & environmental sciences

For a reference copy of the document with all sections, see nature.com/documents/nr-reporting-summary-flat.pdf

Life sciences study design

All studies must disclose on these points even when the disclosure is negative.

Sample size	We demonstrated our virtual birefringence imaging and virtual Congo red staining technique by training a deep-learning model on a dataset comprising label-free tissue sections with a total of 386 and 65 training/validation and testing image patches, respectively, where each image patch had 2048x2048 pixels, all obtained from eight distinct patients. These heart tissue sections were obtained from USC Keck School of Medicine under IRB #HS-20-00151. This sample size aligned well with prior research on similar topics and confirmed to be sufficient by pathologists.
Data exclusions	We did not exclude any data during the analysis.
Replication	We demonstrated our virtual birefringence imaging and virtual Congo red staining technique by training a deep-learning model on a dataset comprising label-free tissue sections with a total of 386 and 65 training/validation and testing image patches, respectively, where each image patch had 2048x2048 pixels, all obtained from eight distinct patients.
Randomization	All training, validation, and testing samples were randomly chosen and allocated.
Blinding	All the evaluation of virtual staining results produced by the deep neural network was blindly performed on tissue images that were not included in the training or validation phases. During the assessment of the staining quality, the three board-certified pathologists were blind to information regarding whether each image was created through the virtual staining technique or the conventional histochemical staining process. Also, the evaluation results of each pathologist were kept confidential from the other pathologists.

Reporting for specific materials, systems and methods

We require information from authors about some types of materials, experimental systems and methods used in many studies. Here, indicate whether each material, system or method listed is relevant to your study. If you are not sure if a list item applies to your research, read the appropriate section before selecting a response.

Materials & experimental systems

n/a	Involved in the study
☒	☐ Antibodies
☒	☐ Eukaryotic cell lines
☒	☐ Palaeontology and archaeology
☒	☐ Animals and other organisms
☒	☐ Clinical data
☒	☐ Dual use research of concern
☒	☐ Plants

Methods

n/a	Involved in the study
☒	☐ ChIP-seq
☒	☐ Flow cytometry
☒	☐ MRI-based neuroimaging
